# Rheological and Mechanical Characterization of Self-Compacting Concrete Using Recycled Aggregate

**DOI:** 10.3390/ma18071519

**Published:** 2025-03-28

**Authors:** Amr ElNemr, Ramy Shaltout

**Affiliations:** 1 Material Engineering at Civil Engineering Department, German University in Cairo (GUC), Cairo 11835, Egypt; 2 School of Energy, Construction, and Environment, Coventry University, Coventry CV1 5FB, UK; ramy.shaltout@coventry.ac.uk

**Keywords:** glass, ceramic waste, self-compacted concrete, fine aggregate replacement, cement replacement, supplementary cementitious materials

## Abstract

Glass and ceramics have a fundamental and crucial role in our lives due to their properties and aesthetic decoration. However, they create serious environmental problems, mainly due to their high occupation of landfills and harmful emissions. Both wastes could be utilized to reduce the natural resources’ adverse environmental effects and exhaustion. With increasing environmental concerns to reduce solid waste as much as possible, the concrete industry has adopted several methods to achieve this goal. Hence, this study examines the performance of self-compacted concrete (SCC) utilizing various percentages of recycled waste materials such as those deposited from glass and ceramic industries. The idea of utilizing recycled waste materials in concrete manufacturing has gained massive attention due to their impressive results in rheological and mechanical states. Recycled glass (RG) and ceramic waste powder (CWP) were utilized to replace fine aggregate and cement, respectively. Five mixes were designed, including the control mix, and the other four mixes had different dosages of RG and CWP as fine aggregate and cement replacement ranging between 5 and 25%. Mixes were tested for both rheological and mechanical properties to evaluate their compliance with SCC requirements as per codes and guidelines. The results revealed that 20% CWP or less as cement replacement and 10% or less of RG as a fine aggregate replacement would provide suitable rheological properties along with mechanical ones. Utilizing recycled glass and ceramic waste powder provides strength similar to the mix designed with natural resources, which helps us keep structures economically and environmentally friendly.

## 1. Introduction

In the last decades, sustainability has been the primary objective of the existing non-sustainable and rapid industrialization for both developed and developing countries. This is especially true with developing countries that adopt vast construction and upgrades to their infrastructure, evolving to produce as much material waste. These material wastes are usually acquired from industrial by-products. Hence, it is difficult to further process them as sustainable materials while experiencing difficulty in their disposal, which concerns vast quantities that increase daily. Most industrial wastes are considered non-sustainable materials. Concrete manufacturing, the second most-utilized material all over the world [1], possesses a higher environmental impact through its carbon footprint. Furthermore, traditional concrete ingredients utilize materials such as cement and aggregate, which, in addition to their manufacturing, possess a carbon footprint as well [2]. Several waste materials such as granite, glass, rubber, ceramic, and marble were suggested as recycled wastes in concrete manufacturing as a fine, coarse aggregate and as a cement replacement in some cases [1,2,3,4,5]. Ceramic waste powder (CWP) is usually generated during manufacturing as a result of defective ceramic products and the shaping of ceramic tiles. Several studies [6,7] explored the utilization of ceramic wastes as a cement, fine, and coarse aggregate replacement. Their results addressed a remarkable percentage of CWP utilization, around 30%.

Most recent work addresses the CWP utilization as a replacement in normal concrete. Few address the CWP utilization in self-compacted concrete (SCC) as a partial substitute for cement since SCC has been developed as a great revolution in the construction industry that requires no external vibration for full compaction. There are various benefits compared to normal concrete, from labor cost reduction to period and design versatility [8]. However, no significant study has been accomplished on CWP as a partial substitute for cement while replacing fine aggregate with glass in SCC [9], despite the huge amounts of other recycled wastes produced through manufacturing and building demolition.

Several studies [4,9,10,11,12,13,14,15,16,17] investigated the utilization of CWP as a cement replacement in SCC to examine the impact at different percentages. Subasi et al. [4] reported that 15 to 20% CWP as cement replacement provided a positive effect on the rheological properties of SCC. Aly et al. [10] observed that limited cement replacement, which ranges between 20 and 30% CWP (by weight), reduced the cement paste/concrete porosity, which in turn enhanced the mechanical and durability characteristics of SCC. As the rheological properties of SCC are influenced by the setting time and hydration process, Li et al. [11] observed that the CWP inclusion increases both the initial and final setting times of SCC. Moreover, Sivaprakash et al. [12] evaluated that the CWP as a cement replacement needs under 14 days of curing while producing concrete with grade M25. Agarwal et al. [6] concluded that increasing the amount of CWP used as cement replacement reduced the decease of concrete density while workability was kept constant. Mohit et al. [13] have explored mortars, not concrete, while utilizing CWP as a partial cement replacement. The results revealed that the mortars incorporating 5% CWP as a cement replacement provided the highest mechanical strength compared to others at 14, 28, 42, and 56 days of acid curing, which is reflected in XRD by the lower intensity of Portlandite (Ca(OH)_2_) peaks than the reference specimen. Their SEM images showed a reduction of pores and voids, confronting the enhancement against an acidic environment. Alsaif [14] concluded that using CWP as a cement replacement had a beneficial influence on most rheological and mechanical properties, specifically workability and compressive strength, and despite the degraded indication of the durability it was concluded that in most cases the material was enhanced. Furthermore, the microstructure of the produced concrete showed a denser matrix. Alternatively, El-Dieb et al. [15] reported the pozzolanic activity of the CWP partial cement replacement as having 80% of its chemical composition contain mainly silica (SiO_2_) and alumina (Al_2_O_3_). Moreover, they addressed the CWP utilization in SCC and recorded their integration to international requirements, while their inclusion enhanced rheological and durability performance at optimized strength in which 40% CWP could be utilized as a partial cement replacement. On the other hand, Chen et al. [16] investigated including the RCA as a replacement of aggregate while replacing the cement with CWP and demonstrated that about 10% to 20% CWP inclusion provided a high strength and enhanced the strength of recycled coarse aggregate (RAC) at 56 days, despite the reduction in the early-age strength of RAC at increased CWP content further than 20%. A range of 10 to 20% CWP enhanced the RAC capillary water absorption and chloride ion penetration resistance, specifically when 60% RCA and 10% CWP were utilized simultaneously, which is attributed to the micro-filler effects and low pozzolanic reactivity of CWP. Similar findings were deduced by El-Dieb and Kanaan [9] and Mohit and Sharifi [17] who added that 10% to 20% CWP is more effective for cement substitutes: 10% for early ages, and 20% for later ages. Further, Kannan et al. [18] recommended the inclusion of CWP in a range of 10 to 40% as a partial cement replacement, as it would provide high-performance concrete and excellent durability. Nevertheless, the attitude towards the improvement was explored by microstructure, which ensured no significant effect on cement hydration and confronted the creation of dense packing particles.

On the other hand, several researchers [19,20,21,22,23,24] recommended the utilization of glass as a replacement for concrete ingredients, especially fine aggregate as it provides a unique performance. In addition to the fine aggregate replacement, the privileges of glass from having thermal and ecological impacts on the environment were proven when used as lightweight brick as stated by Ketov et al. [25]. In addition, there are the ecological advantages compared to raw materials, where the mining process provides emissions while recycled waste requires some treatment process like crushing, etc. Shayan and Xu [19] stated that the mixed color glass in concrete could be used as coarse aggregate (4.75–12 mm), fine aggregate (0.15–4.75 mm), or as an SCM when ground to form glass power (<10 mm) as a partial cement replacement. Hamada et al. [20] explored the utilization of waste glass aggregates (WGA) and their influence on concrete performance. Their observation summed up that several factors could influence the mechanical qualities and durability of WGA concrete such as size, type, replacement ratio, mixing method, and curing condition. The main reasoning for use was the WGA’s refined pore structure and densified microstructure. Their results revealed that the best performance was when utilizing the WGA as a fine aggregate replacement; however, the optimum replacement levels varied from 20 to 30% for fine aggregates and 10 to 20% for coarse aggregates, with an indication of lighter-weight concrete than natural aggregate. Moreover, Sharifi et al. [21] incorporated recycled glass as fine aggregate in SCC concrete to assess the rheological properties, strength, and durability in five different portions.

There has been further investigation of combining recycled wastes as a replacement for coarse and fine aggregate in addition to cement replacement. For instance, Kou and Poon [22] explored the rheological and mechanical properties of the SCC-incorporated recycled glass (RG), less than 5 mm in size, along with crushed granite of a maximum size of 20 mm. They concluded that RG is economically friendly, eco-friendly, and structurally friendly, while providing strength at acceptable limits by code and guidelines. Srikanth and Lalitha [23] prepared SCC utilizing several percentiles of WGA as a partial substitution of fine aggregate. Their results observed that the optimum WGA replacement to satisfy the SCC rheological properties was 30%, while for best performance in mechanical properties it was 35% of cement replacement combined with 30% of fine aggregate replacement. Finally, Singh and Siddique [24] incorporated metakolin along with RG into 24 different SCC mixtures. Slump flow, V-funnel, L-box, and U-box tests related to SCC’s passing and flowing ability were enhanced as the CRG content increased in the mix; however, mechanical properties were decreased. Thus, substituting the cement with MK in CRG-incorporated SCC mixtures improved the strength properties at all of the glass replacement levels.

Several research studies were carried out to observe the effect of various recycled and waste materials in manufacturing self-compacted concrete executed in many countries. Multiple replacements of recycled and waste materials with all-natural concrete ingredients existed. Hence, the significant effect of CWP and RG on SCC performance with the recommendation of RG as a fine aggregate replacement is deduced from the previous studies. Thus, this study aimed to discover the impact of CWP on SCC production as a partial cement replacement while combining glass as a fine aggregate replacement. The CWP was replaced by 15, 20, and 25%, with a gradual replacement of the fine aggregate by 5% by weight reaching 15%.

## 2. Research Significance

Recently, the ecology of construction materials, specifically concrete, has paid great attention to environmental friendliness, eco-friendliness and sustainability. This yet requires in-depth investigation and a variety of studies in different concrete technologies exposing innovative ideas such as utilizing construction wastes in concrete manufacturing: reducing the disposal of wastes, recycling the wastes, and reducing the deployment of natural strategic resources while maintaining a suitable carbon footprint lower than that possessed by concrete manufacture. Several studies have investigated the utilization of recycled wastes as fine and coarse replacement or as cement replacement. Most of these investigations focused on the usage of these recycled wastes in conventional concrete, not SCC ones or special concrete types, recommending the optimized values of replacement for utilizing these wastes individually, as in CWP for cement replacement and RG for fine aggregate replacement. The judgment mainly focuses on hardened concrete properties. Very few studied the combination of recycled wastes, and yet no special types of concrete as SCC. In this study, the focus is on the performance of self-compacted concrete, which is a special concrete type, while utilizing the recycled wastes of two different materials, CWP and RG, for cement and fine aggregate replacement. The recommended percentage for individual utilization of CWP and RG as cement and fine aggregate replacement ranged between 10 and 25% for CWP and from 10 to 30% for RG, as per the literature review. Thus, the performance of combining both CWP and RG as cement and fine aggregate replacement is measured in terms of rheological and hardened properties. The results showed a unique influence with optimized values which were not the same as those recommended when utilized individually.

## 3. Materials and Methods

**Figure 1:**
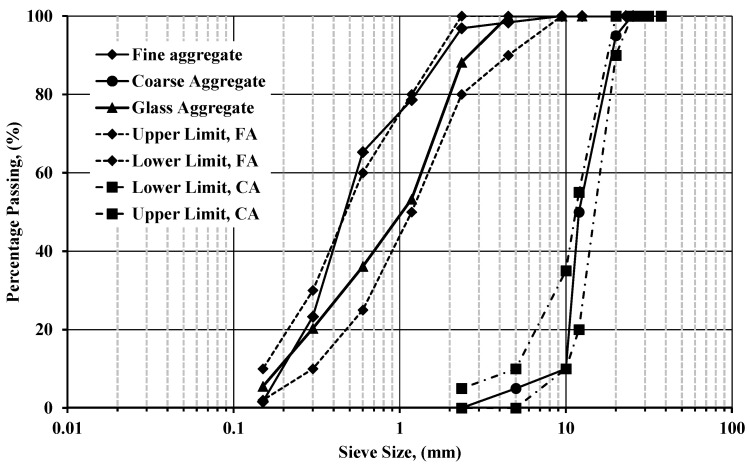
Shows the sieve size grade distribution of coarse, fine, and glass aggregate [27].

In this study, five mixes, including the reference mix, were designed to investigate the performance of the self-compacted concrete using recycled waste powder as supplementary cementitious materials, such as ceramic powder, and recycled aggregate such as fine glass aggregate. Table 1 shows the mix design portions after many trial batches to maintain the favorable rheological properties of self-compacted concrete. The control mixtures were created with 100% fine aggregate (FA) and cement (C). The other mixes 1 and 2 had constant cement replacement by 20% CWP and varied fine aggregate replacement with glass aggregate of 2.36 mm, as shown in Figure 1, for 5 and 15%. Mixes 3 and 4 were replaced with a constant 10% fine aggregate with glass and the various percentages of cement replacement by 15 and 25% CWP. The variation was selected based on the existing literature, which stated that 20% of CWP as cement replacement would be the optimum solution [4,9,10,11,12,13,14,15,16,17], and 10% of glass as a fine aggregate replacement would be the optimum as well [19,20,21,22,23,24]. It should be mentioned that the water-to-cement ratio (*w*/*c* ratio) was 0.42 at mixes. The control mix was based on the study carried out by Mohammed [26] mentioned earlier, as it provides good results using similar materials existing in the Egyptian market.

### 3.1. Cement

According to the data provided by the manufacturer, the cement type used here in this study was of grade 42.5 with typical reactivity, donated by the letter “N”. The datasheet provided the cement’s physical, chemical, and mechanical properties. Table 2 provides the chemical composition of cement, ceramic waste powder, and recycled glass. Finally, the air Blaine fineness for the cement was 3780 cm^2^/kg and a specific gravity of 3.15.

### 3.2. Ceramic Waste Powder

Ceramic waste powder (CWP), produced from the final polishing process of ceramic tiles, is produced in significant amounts and is composed mainly of silica and alumina; hence, it has the potential to be used as supplementary cementitious material as an alternative to cement. This investigation produced ceramic powder (CWP) by crushing it into pieces through the Los Angeles apparatus or laboratory grinding machine. Then, the ceramic was ground to be smaller than 600 μm (passing sieve No. 30, size 600 μm) in diameter, as shown in Table 2. The chemical composition of ceramic and cement and the high percentile of silica and alumina reached 63.9% and 18.29%, respectively. The ceramic was used as cement replacement in three percentiles, 15, 20, and 25%. The percentage was selected in the existing literature, indicating that the CWP could provide high durability resistance and strength if optimized at 20% at a late age [4,9,10,11,12,13,14,15,16,17].

### 3.3. Fine and Coarse Aggregates

Figure 1 shows the grain size distribution of coarse, fine, and glass aggregates and the upper and lower bounds of ASTM C33 [28]. The natural coarse aggregate used here in this study was a nominal maximum aggregate size of 25 mm. The specific gravity of coarse and fine aggregate was valued at 2.50 and 2.55, respectively. The absorption and moisture content of the fine and coarse aggregate used was 1.21, 0.6, 1.20, and 0.36%. The crushing and impact values of coarse aggregate would equal 20 and 14.8%. All tests are handled per the Egyptian code for designing and implementing concrete installations—Manual of testing, ECP 203 [29], and BS 882 [30], as noticed.

### 3.4. Superplasticizer

Sikament^®^ NN is a highly effective admixture or superplasticizer for concrete that is especially suitable for producing free-flowing and high-strength concrete, especially in hot climatic conditions, with very high plasticity and good slump-keeping properties for concrete. The chemical admixture was provided by Sika INC (Cairo), Egypt and named Sikament-NN. Its chemical basis was modified polycarboxylate as ASTM C494 [31] and ASTM C1017 [32]. Its occurrence in many different works proves its enhancement to construction structures by reducing the water by 20% and by the ability to place slender components with dense reinforcement and increase the compressive strength.

### 3.5. Glass

Like ceramic powder, many researchers [19,20,21,22,23,24] denoted that glass could provide better performance in strength and durability, especially sorptivity and water absorption, in addition to the thermal insulation that could be provided as stated by Ketov et al. [25]. Glass could be recycled from crushed beverage bottles, broken windows, door panels, etc. Due to its chemical composition of high silica, it has hydrophobic action that repels any reaction with water, which raises the possibility of having an ASR (Alkali Silica Reaction). This action could occur between waste glass and alkalis produced by cement during hydration, lowering concrete strength and durability. Then, it leads to cracks in the concrete. However, this reaction could be mitigated using ASR suppressants by other materials such as fly ash or other recycled wastes as supplementary cementitious materials as with CWP. Experimental evidence proved that the potential problem of this reaction could be decreased with reduced glass aggregate particle size, revealing that glass aggregate less than 2.36 mm would effectively minimize ASR expansion and assure pozzolanic activity. Thus, in this study, the glass purchased was composed of several broken sheets from different works. The Los Angeles laboratory grinding machine ground the glass into smaller particles. Then the crushed particles were collected for sieve analysis distribution to ensure the largest passing percentile would be around 2.36 mm sieve diameter. Figure 1 shows the sieve size distribution of the glass used as a partial replacement of sand at 5, 10, and 15% based on the collected literature review. The figure shows that the glass and fine aggregate distribution was within the upper and lower bound assigned by ASTM C33 [28]. It should be mentioned that the glass particles were angular in shape and rough in texture.

## 4. Specimens Preparation

**Figure 2:**
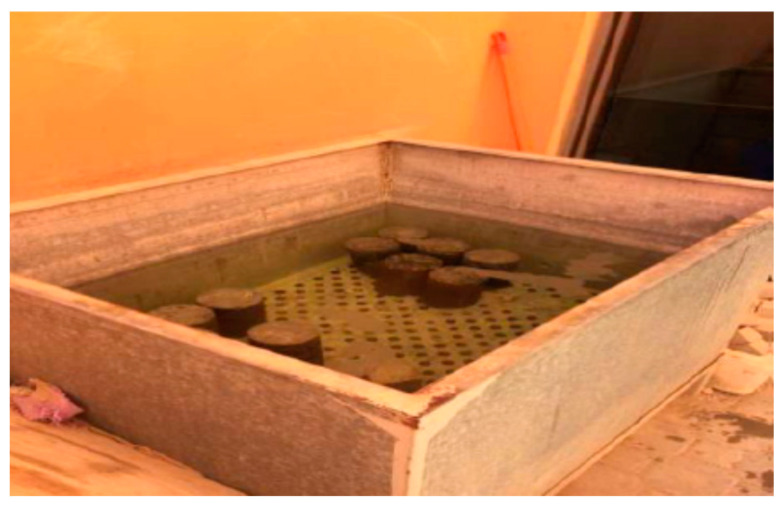
Shows the curing tank for the specimens.

A total of 30 cube specimens dimensioned 150 mm, 15 cylinder specimens of diameter 150 mm and height 300 mm, and 15 prism specimens of length 500 mm with height and width of 100 mm, were cast. The cube, prism, and cylinder specimens were utilized for evaluating the compressive, flexural, and splitting tensile strength, respectively. The molds were adequately cleaned and tightened to keep exact dimensions during casting. The molds’ inner surfaces were coated with a thin oil film to facilitate demolding after concrete hardening. The fresh concrete was cast in molds without any external work. The specimens were demolded after 24 h from the cast and marked concerning the mix symbolization. The specimens were cured at an ambient room temperature of 21 ± 1 °C for 7 and 28 days in the curing tanks, as shown in Figure 2. It should be mentioned that for each mix, three cube specimens were tested at 7 days and the other three were tested at 28 days along with the prism and cylinder specimens.

## 5. Testing Method

The tests for the self-compacted concrete here took two phases: phase I at the rheological state and phase II at the hardened state after curing. Phase I consists of test sets for SCC: slump flow, slump T_500_, J-ring, V-funnel, V-funnel T_5 min_, and L-box. The tests were handled according to ASTM C1611 [33], C1621 [34], EN 12350-9 [35], and EN 12350-10 [36]. The upper and lower limits for each rheological test are addressed in Table 3 as per ECP 203 [29].

The rheological test can be explained and illustrated as follows:

(a) Slump flow test

**Figure 3:**
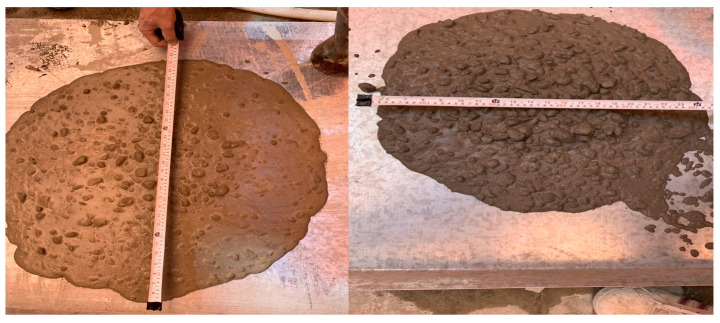
Shows the slump flow diameter measurement.

The test is handled through ASTM C1611 [33] which states that the sample of fresh concrete should be placed into the frustum on the rigid plate. The frustum is then removed so that the freshly mixed concrete can flow into a diameter range between 600 and 800 mm as assigned by ECP 203 [29] and presented in Table 3. Figure 3 shows the determination of diameter by measuring the slump flow diameter perpendicular to each other and getting the average diameter through the below Equation (1):

(1) $$D_{slump\;flow}=\frac{D_{1}+D_{2}}{2}$$

where *D*_1_ and *D*_2_ are the diameters of the slump flow for SCC crossing each other perpendicularly.

(b) Slump flow time at T_50 cm_

**Figure 4:**
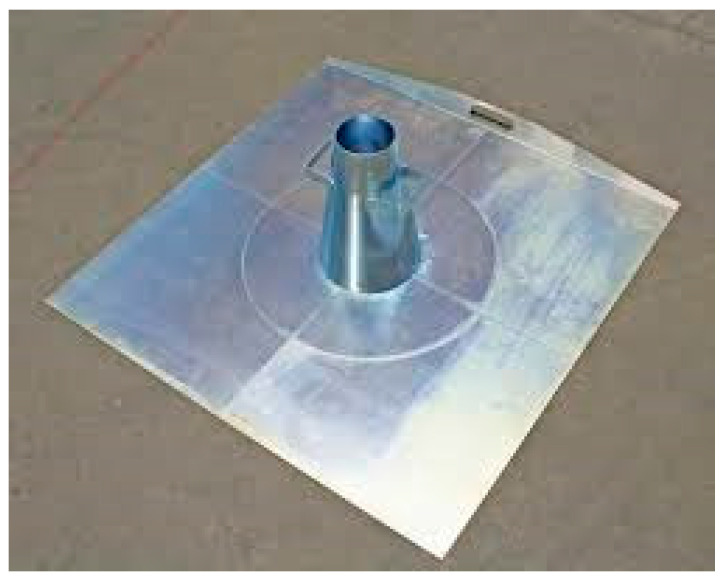
Shows the slump flow of an engraved diameter of 500 mm at which the time is measured.

Similar to the previous test, fresh concrete is poured inside the frustum which is placed on the rigid plate with the engraved indication for a diameter ring of 500 mm, as shown in Figure 4. Then, the frustum is removed while the stopwatch is counting the time elapsed for the slump flow to reach a diameter of 500 mm in seconds [34]. The ECP 203 [29] assigned a range of 2 to 5 s.

(c) J-ring flow

**Figure 5:**
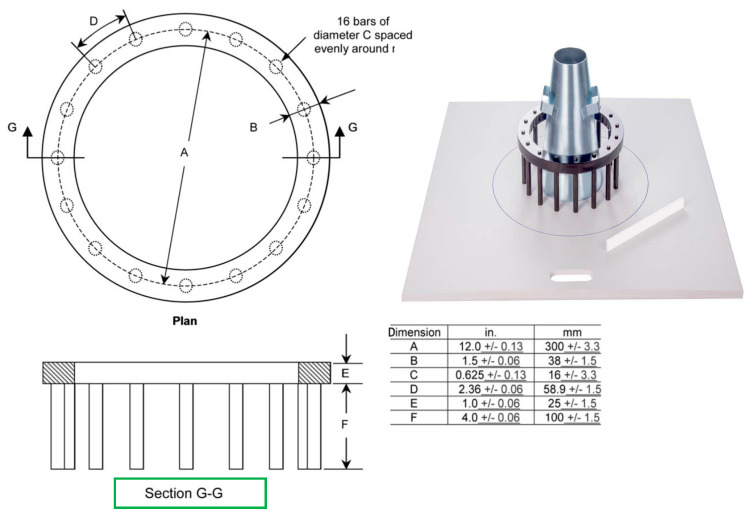
Shows the J-ring with details of spacing and reinforcement diameter [34].

The test examines the ability of concrete to pass (pass ability) through a reinforcement diameter of 16 mm and spacing of 59 mm, see Figure 5. The selection of diameter and spacing of 16 mm was based on the average sizing of bars as per ASTM C1621 [34]. The slump flow through the J-ring is calculated by measuring the two diameters crossing each other perpendicularly and getting the average between them as the following Equation (2).

(2) $$D_{J-ring}=\frac{D_{1}+D_{2}}{2}$$

The ECP 203 [29] defined the upper and lower limits for the diameter as the fresh concrete passing the reinforcement with a margin of a 0 to 20 mm increase in diameter (300 + 0 or 300 + 20), as illustrated in Figure 5.

(d) V-funnel

**Figure 6:**
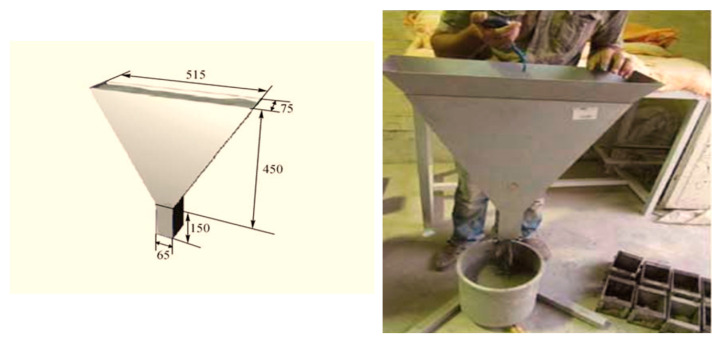
Shows the V-funnel dimensions and the method of testing. (dimensions in mm).

The test starts using the V-funnel as shown in Figure 6. The V-funnel has dimensions of 515 mm and 75 mm with a height of 450 mm and a rectangular end of 65 and 75 mm; see Figure 6. This rectangular end has a sliding gate that opens when the V-funnel is filled where a cylinder is placed to be filled with fresh concrete. The time elapsed for falling the concrete into the cylinder is measured using a stopwatch. The ECP203 [29] assigned a margin of 6 to 12 s for the concrete to fill in the cylinder and fall entirely from the V-funnel. This elapsed time is denoted by (t_o_). The idea of the test is to evaluate the flowability of concrete continuously and to ensure that no blockage could occur while casting the concrete. In addition, the concrete amended the required viscosity and filling ability as self-compacting concrete [35].

(e) V-funnel after 5 min

Similar to the testing assigned above, the V-funnel can undergo another method of testing by leaving the freshly mixed concrete in the funnel for 5 min after cast and before opening the gates; see Figure 6. Then, the free fall of the mixed concrete into the cylinder would provide the suitability of this mix for being SCC without causing blockage or missing the necessary viscosity after the start-up of the hydration process for 5 min. The test examines the period required for filling the formwork after initiating the hydration process towards the initial setting time, which in this case would be heavily filling the voids and passing through reinforcement shaping the mold or formwork [35]. The time elapsed is measured using the stopwatch and the limits amended by ECP 203 [29] are calculated from (t_o_) since it was measured in the previous step to (t_o_ + 3), as shown in Table 3. It should be recalled that the limit of t_o_ is between 6 and 12 s which means that the limit in V-funnel testing after 5 min is between 9 and 15 s [29].

(f) L-box test

**Figure 7:**
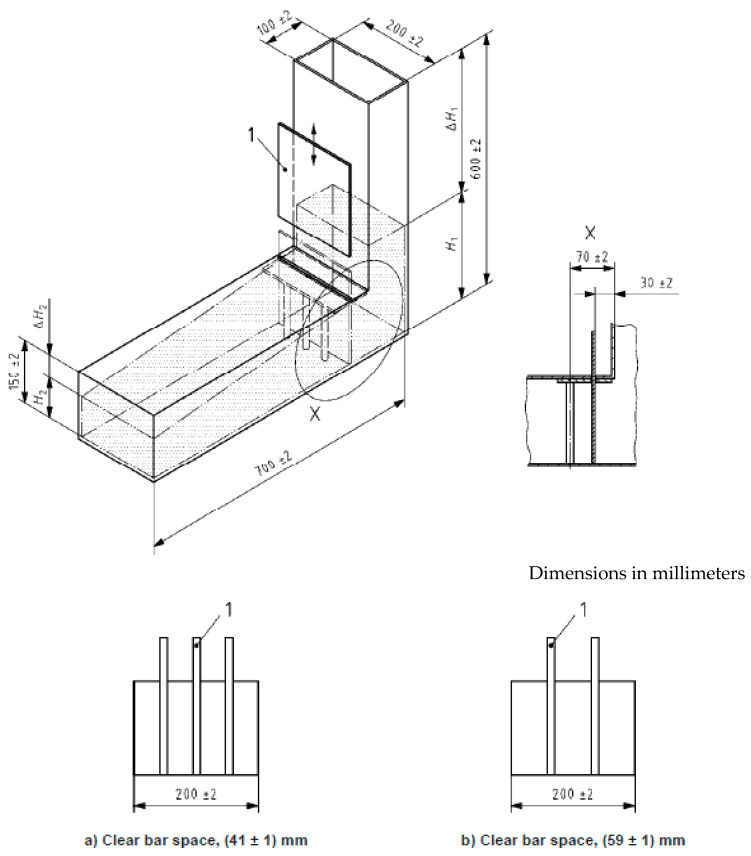
Shows the L-box dimensions and the two sets of reinforcement spacing at (**a**) 41 mm, and (**b**) 59 mm [36].

The idea of the L-box test is similar to the that of the J-ring, and it is main purpose is to evaluate the passing ability of SCC through passing the concrete’s weight through tight openings including congested reinforcement at different spacing. The test is amending only to spaces on 41 mm with three reinforcement bars, and the other with 59 mm between bars only, as shown in Figure 7. A ratio is measured between the vertical section height (*H*_1_) and the height of concrete at the horizontal section *H*_2_ as clarified in Figure 7. Equation (3) shows the ration calculation under the name of “PL”, which represents the passing or blockage behavior of SCC [36].

(3) $$PL=\frac{H_{2}}{H_{1}}$$

where, *H*_1_ represents the heights of the concrete in the vertical section, while, *H*_2_ measures at the end of the horizontal section, see Figure 7. Nevertheless, the ECP 203 [29] ranged this ratio between 0.8 and 1.0. The selected gate in this research was the one with two reinforcement rebars with spacing of 59 mm, to compile with the results of those in the J-ring slump flow test.

**Figure 8:**
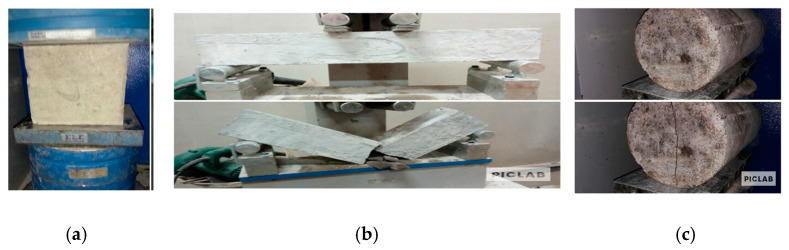
Shows the testing of the cube, prism, and cylinder specimens for (**a**) compressive, (**b**) flexural, and (**c**) splitting tensile strengths.

Then in phase II, the specimens were dried in the laboratory and tested through various applied test equipment, as shown in Figure 8. The test was typically handled on cube specimens during testing in compression into the universal testing machine of capacity 2000 kN for mechanical properties evaluation, see Figure 8a. The cube specimens were tested at a pacing rate of 240 kg/cm^2^ per minute according to EGP 203 [29] until the specimens failed. Meanwhile, prism specimens were tested at a pacing rate of 24 kg/cm^2^ per minute by implementing compression loading on the prism specimens’ longitudinal direction, as shown in Figure 8b. Finally, cylinder specimens were tested in compression longitudinally at a pacing rate of 12 to 24 kg/cm^2^ per minute as clarified in Figure 8c for measuring splitting tensile strength. Recall, the compressive strength was evaluated at 7 and 28 days of age, while the flexural and splitting tensile strengths were measured at 28 days only.

## 6. Results and Discussion

The following section presents the fresh state properties.

### 6.1. Fresh State Properties

As stated earlier, five tests evaluate the fresh properties in this study. The results were obtained and compared by the EGP 203 [29] as follows:

#### 6.1.1. Slump Flow

**Figure 9:**
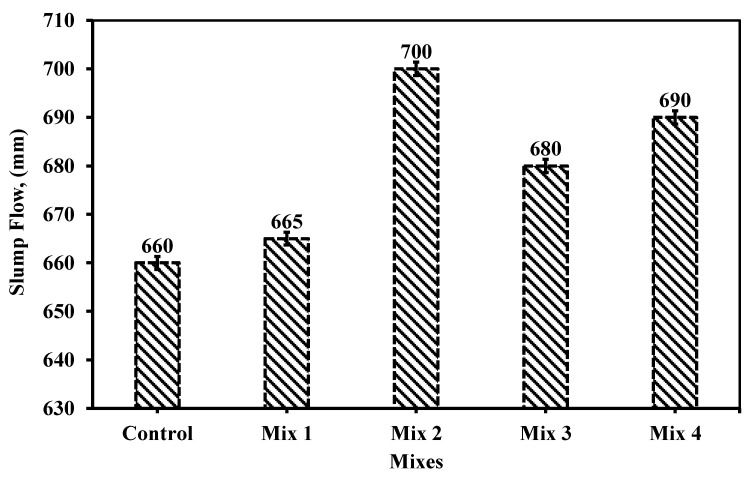
Shows the slump flow measurement for the five mixes.

Figure 3 shows the measures of the *D*_1_ and *D*_2_ for evaluating the average diameter of the slump flow. As clear from the figure, the mix showed a high flowability. Figure 9 shows the slump flow value for the five mixes. The figure shows the slump flow between the limits set by ECP 203 [29]. The limits are between 600 and 800 mm. The maximum value was achieved using 20% CWP as cement replacement and 15% RG as fine aggregate (mix 2). The slump flow provides a value of 700 mm. While the minimum value provided was the control mix reaching 660 mm. The results showed that using 5%, RG would not increase the flowability significantly as in mix 1. However, increasing the percentile of replacing the fine aggregate increased the flowability. Similarly, increasing cement replacement by CWP from 15% to 25% at constant fine aggregate replacement by 10% RG increased the slump flow by 1.5%.

Comparing the slump flow of each mix, in mixes 1, 2, 3, and 4 with the control it was found that the enhancement in the slum flow was 1, 6, 3, and 5%, respectively. This provides that replacing 15% RG with fine aggregate and 20% CWP replacing cement would give better high range flow behavior than the other percentiles. Thus, increasing the utilization of the recycled wastes would in turn enhance the flowability of SCC relevant to the other SCC tests.

Singh and Siddique [24] have studied the use of crushed glass as a finer aggregate replacement while cement was replaced by MK. Based on their results, which ranged from 600 to 750 mm, the slump flow showed an increase in their flow as long as the fine aggregate replacement by CRG increased; however, when replacing the cement by MK, the slump flow was significantly influenced and was lowered, which complies with the results here in this study. This finding was confirmed by Ali and Al-Tersawy [37] when they explored replacing fine aggregate with crushed glass only. Likely, Jassam et al. [38] observed that at the RG content increase as fine aggregate replacement, the slump of the traditional normal concrete decreases, but it must be noted that the utilization was in normal concrete not SCC. The results showed that slump flow grew higher and increased as the fine aggregate replacement with CRG increased, although the increase of binder powder by 50 kg/m^3^ or a constant 10% of SF [39]. It should be mentioned that the ranges were between 640 and 880 mm.

#### 6.1.2. Slump T_500_

**Figure 10:**
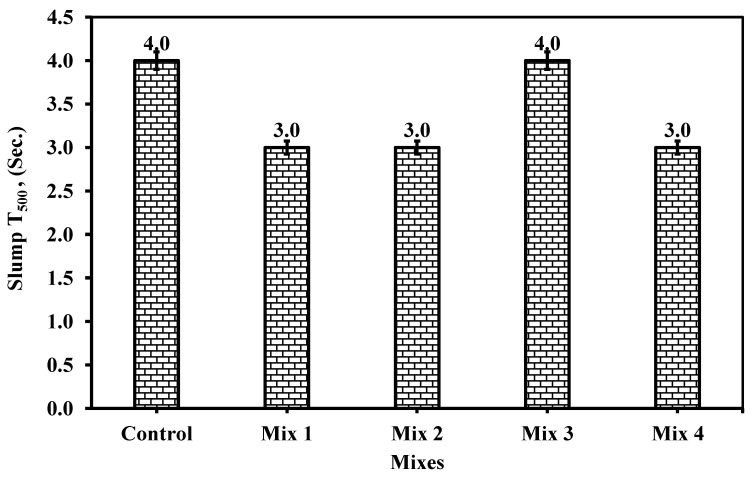
The slump T_500_ measurement for the five mixes.

As shown in Figure 10, the time required to spread the mix reaches 3 s at minimum and 4 s at maximum. ECP 203 [26] limits the slump diameter to 500 mm (50 cm) between 2 and 5 s. Thus, all mixes including the control already satisfy the limits.

From the figure, control and Mix 3 (15% CWP as cement replacement and 10% RG as fine aggregate) possess the highest flow time among the other mixes, and thus low flowability, which means high viscosity might be encountered in these two mixes. Increasing the cement replacement by CWP to 20% while increasing the fine aggregate replacement by RG from 5 to 15% would keep flowability constant. In other words, changing the fine aggregate replacement by RG would not affect the slump flow at a diameter of 50 cm. In contrast, mix 3 reduces the cement replacement by 5% CWP while increasing the GR as a fine aggregate replacement to 5% from that of mix 1. Thus, as shown in mix 4, when increasing the cement replacement by 25% CWP at 10% RG fine aggregate replacement, the slump flow changes against flowability as in mixes 1 and 2. Thus, it is usually the number of fines that influence the self-compacting behavior of these mixes. The CWP as a cement replacement would enhance this self-compacting concrete.

The time elapsed for each mix was nearly reduced by 25% than that of the control mix except for mix 3. Mix 3 possessed the same time, reaching the 50 cm engraved diameter spread in 4 s. Thus, increasing the RG only is not the prime factor affecting the flowability but also increasing the powder content with sizes as in CWP would help more in the flowability of the concrete mixture and ensure the viscosity towards binding the concrete components together. Mix 3 exhibited a mixture of 15% CWP and 10% RG replacement of cement and fine aggregate which represents the relevant ratio of 1.5 to 1 while the relative ratio of mixes 1, 2, and 4 was 4:1, 1.33:1, and 2.5:1. Thus, it seems the ratio of 1.5:1 would be the controllable ratio for achieving similar slump flow in the behavior of traditional concrete ingredients.

No results provided the time for slump flow at 500 mm diameter (T_500_). In this study, the test was performed and achieved a similar trend to the original slump flow. It is recommended to further investigate this test in the rest of the literature review, as shown from Figure 10. The test results revealed that the period to fulfill a diameter of 500 mm took a range of 3 to 4 s, which is considered within the acceptable range which was as per ECP 203 [29] ranging from 2 to 5 s.

#### 6.1.3. J-Ring

**Figure 11:**
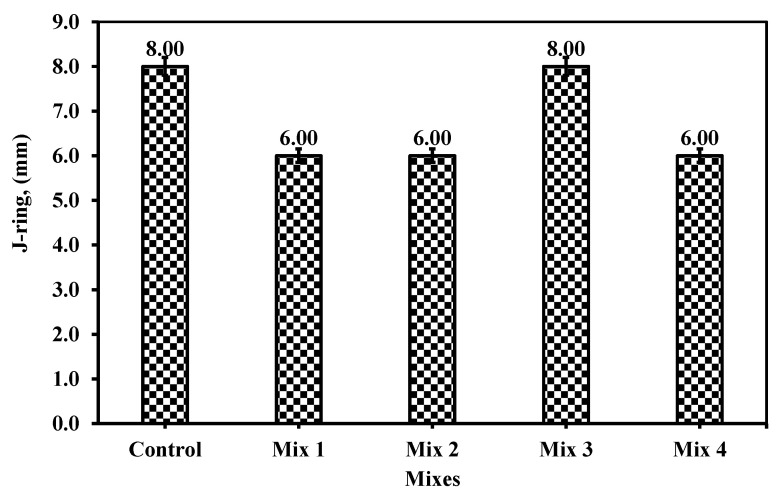
The J-ring measurement for the five mixes.

The J-ring is a very crucial issue when it comes to reinforcements. The idea of self-compacted concrete incurred the flowability of concrete through the congested reinforcement. According to ECP 203 [29], the concrete should flow through the reinforcement at a diameter between 0 and 20 mm exceeding the ring diameter (of 300 mm). From Figure 11, all the mixtures range in diameter from 6 to 8 mm over the ring diameter which is considered within the limit and passes through the spacing of the reinforcement. Similarly, to the Slump T_500_, the results confirmed a similar trend to those of slump flow and slump T_500_. From the literature review, none of the studies contribute to the passability of concrete through the J-ring while replacing fine aggregate with glass or cement with any additives or recycled wastes. Thus, the J-ring showed the blockage that could occur with a bar of diameter of 16 mm. Nevertheless, the mixtures took nearly 8 s maximum for mix 3 to pass through reinforcement, which is noted to be the least mix among the examined mixes. The acceptable range as set by ECP 203 [29] is between 0 and 20 mm which indicates that the SCC mixture would be acceptable if it is just past the ring while the obstacles exit or exceed 20 mm more than the ring’s diameter (300 mm); see Figure 11, of obstacles passing through the spacing of their reinforcement (300 ± 20 mm). Thus, the mixture showed unique uniformity and highly spread through obstacles as the congested reinforcement. These results provide an optimistic influence to implement these mixes even at highly congested reinforced structural elements. The values of the control mix, mix 1, 2, 3, and 4, are 8, 6, 6, and 8 mm. The behavior was reversed when it comes to passability due to the presence of high CWP content in most mixes, except mix 3. The passability of the concrete mixtures was influenced, and a smaller diameter exceeding the ring’s diameter was observed, as shown in Figure 11. Among the mixes, mix 3 is relevant to the control one. The reduction in the passability and the flowability through the spacing between the reinforcement was observed by 25% in mixes 1, 2, and 4. Mix 3 behaves similarly to the control mix, which is the SCC mixture. Thus, it seems relevant to the previous results obtained in the slump flow of T_50 cm_ indicating the importance of the optimized recycled waste replacement between the cement and fine aggregate. Thus, the measuring of the powder effects should be considered an important aspect in the SCC especially when it comes to two different recycled wastes being utilized.

#### 6.1.4. V-Funnel

**Figure 12:**
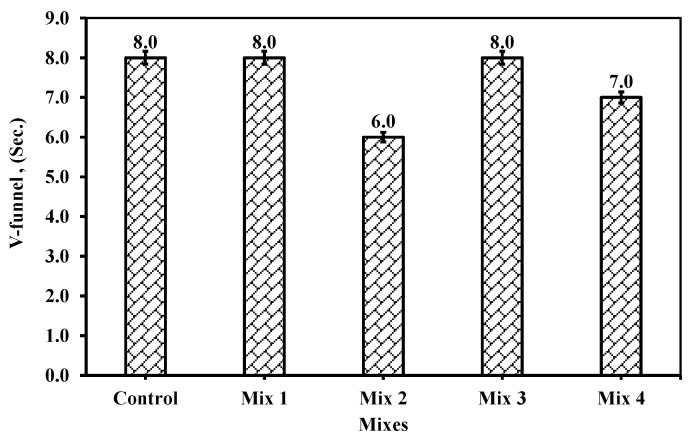
The V-funnel measurement for the five mixes.

Figure 12 shows the V-funnel for each mix, including the control one, in seconds. The V-funnel measures the period of mix flow after filling the funnel. From Figure 12, Mix 2 provided optimum values among the mixes, as the time for the mix to flow after filling the V-funnel took just 6 s, which is the filling range adopted by ECP 203 [29] of 6 to 12 s. Nevertheless, all mixes fell within the range of 6 to 12 s. Mix 4, where the cement replacement reached 25%, slightly influences the V-funnel for a slight enhancement by 12.5% relevant to the other mixes.

None of the literature reviews mentioned which type of V-funnel testing would be considered, though it is understandable that only the period for V-funnel ingredients to flow was evaluated. The statistical data in the literature review handled by Revilla-Cuesta et al. [40] showed that it would be better to replace fine aggregate with RCA, as it provides a lower period for the V-funnel than coarse aggregate replacement, even if used as 100% fine aggregate replacement. On the other hand, Srikanth and Laritha [23] reported that the increase of fine aggregate replacement with crushed glass increased the flowability of the mix and reduced the time required. The optimum percentage was reached at 40% and provided 8.7 s on V-funnel testing, which was considered within the limit (6–12 s) and recommended by codes [29].

However, the mixture ingredients contain fines such as fly ash, which replaced cement by 15%, and alccofine, which replaced 20% of cement. In other words, only 65% cement of the total binder were included. It should be noted that fly ash improves the slump and workability due to the spherical shape of its particles. El-Dieb and Kanaan [9] recommended or conducted a range of 10 to 20% of CWP as cement replacement so that the slump can be influenced positively. Any more than this percentile and the slump would be reduced, and the mixture would become stiffer than the reference mixture. From the results, the increase of RG replacing fine aggregate at 20% CWP in the mixture content, as in mix 2, showed that less time elapsed and less blockage occurred. This behavior is attributed to the smooth surface of the fine glass particles that might helped in sliding the CWP and cement particles over each other even with their high fineness degree (<75 µm). Mix 4 showed a lower time elapsed than the control mix, and mixes 1 and 3 for the flow of the concrete mixture without blockage. Further investigation is required to prove this hypothesis through image processing that could handle the movement of particles while falling into the cylinder and justify the sliding theory between particles. On the other hand, handling other tests could be suggested to measure the viscosity of the concrete mixture produced, which might impact the time elapsed while filling the cylinder from the gate of the V-funnel.

#### 6.1.5. V-Funnel After 5 min

**Figure 13:**
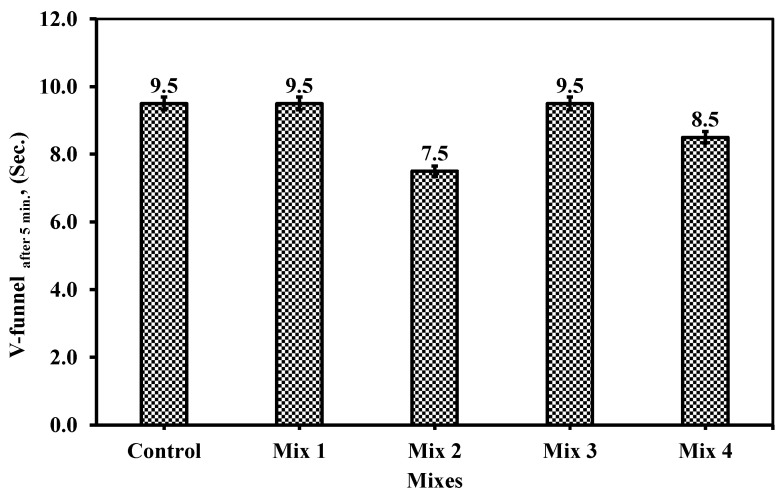
The V-funnel measurement after 5 min for the five mixes.

Increasing in accuracy, the test was handled again, but after leaving the cast poured onto the V-funnel for 5 min and then opening the flow of concrete, consider the actual cast process that happened or occurred inside. Although the results of the testing did not differ from those of immediate testing V-funnel the results revealed a shorter period of seconds than the usual V-funnel testing after 5 min. The period for V-funnel flow was increased by 1.5 s for each mixture showing consistency, viscosity, and homogeneity of the mix. Figure 13 shows the V-funnel for each mix, including the control one, in seconds.

With similar results to the used V-funnel from the previous result, reduction and enhancement between the mixes were achieved. Many studies discussed the SCC concrete using recycled waste. However, limited studies, or nearly none of them, discussed the V-funnel after 5 min of cast, despite its importance in actual application.

These results confirm the viscosity provided by mix 2 and then 4, in which the glass powder is about 15 and 10% of the total fine aggregate content while CWP ranges from 20 to 25% as cement replacement. Thus, it provides similar optimal values with ECP 203 [29] code limits ranging between t_o_ (usual V-funnel time) and t_o_ + 3 s, which was achieved by the mixes.

Mix 2 and mix 4 exhibited a reduction in the time elapsed compared to that of the control mix of 25 and 12%, which is similar to the V-funnel test results. The V-funnel, after 5 min, would measure the flowability of the concrete mixture in its fresh states after leaving the mix in the V-funnel for at least 5 min to ensure the initiation of the hydration process, which in turn solidifies the movement. This represents the actual field construction for spreading the concrete into the formwork which might take longer in time.

The trending behavior in the V-funnel after 5 min indicates that the replaced material, whether the CWP or RG, acted as filler rather than binding materials, or might require activation through adding another component to the concrete mixtures. Mix 4 showed less time elapsed than the control mix and mixes 1 and 3, but higher than mix 2, though the CWP content represents the highest content (25%). On the other hand, mix 2 showed the lowest time elapsed while the CWP did not exceed 20%, which confirms the hypothesis of recycled materials acting as filler rather than reactive binding material contributing to the hydration process. Thus, it can be concluded that the CWP and RG did not contribute to the hydration process or activate through the elevated temperature when cement hydration was initiated.

#### 6.1.6. L-Box

**Figure 14:**
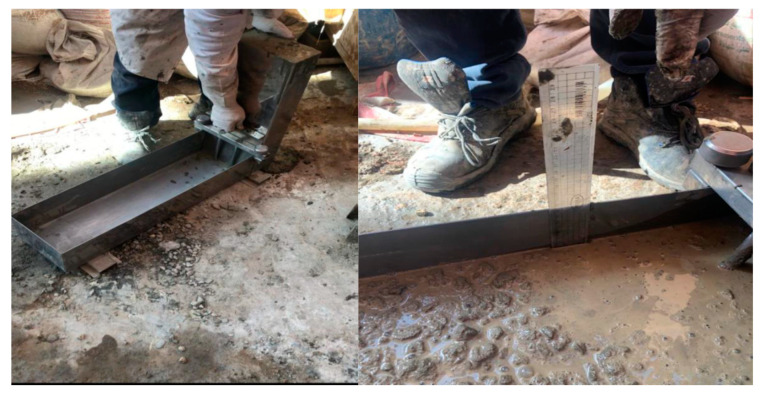
Shows the measuring of the height H_1_ and H_2_ to determine the ratio for passability of the concrete mixture through L-box and spacing of the reinforcements at 59 mm.

**Figure 15:**
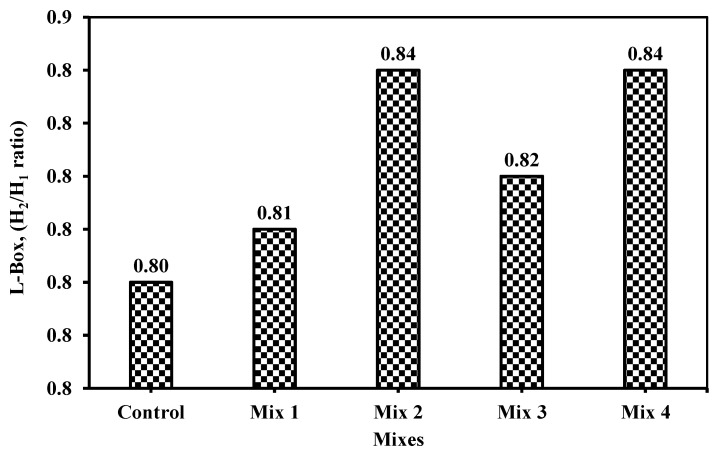
The L-box measurement for the five mixes.

This is the last test recommended by EN 12350-10 [36] and ECP 203 [29] when discussing SCC. The L-box is a representation of the flowability through reinforcement for concrete. It measures not only the flowability but also the passability of concrete, as shown in Figure 14 in which the heights at the vertical and at the end of the horizontal were measured. It is used for utilizing concrete underwater as per the Japanese design code. However, as per ECP 203 [29], it is one of the six tests that should be checked when addressing SCC. The limited range, according to ECP 203 [29], was from 0.8 to 1.0. Figure 15 shows the result of the L-box ratio for the five mixes. The results for all mixes past 0.8 or equal to 0.8 as in the control mix. In contrast to the other tests, increasing the percentile or ratio indicated good passability and flowability of concrete mix. It ensures the filling ability of the box.

From the literature review [37,39,40,41], it can be deduced that studies explored the L-box in SCC which indicated the lower importance of the slump flow, V-funnel, and J-ring were justified. Ali and Al-Tersawy [37] observed a higher ratio at high glass powder replacement of fine aggregate, reaching 50% in their three group sets and, thus, stated that the passability and the flowability could be considered at their peak if the glass powder replacement reached 50% of the total fine aggregate. Similar results were deduced by Revilla-Cuesta et al. [40] through their statistical data collection using RCA (recycled coarse aggregate) as a replacement for coarse and fine aggregate. They denoted that if fine aggregate replacement increases, the L-box ratio increases, even though coarse aggregate was replaced by RCA. Most data recommended 50% to 50% percentile replacement for both fine and coarse aggregate combined, or a 20% replacement of fine aggregate would provide the highest ratio for L-box among the other collected data tests (i.e., 0.97) [41]. On the other hand, Singh and Siddique [24] worked on SCC using metakaolin recycled glass which is considered to be similar to this study, and found that L-box increases with increasing fine aggregate replacement by GP reaching 50%, and decreases when increasing the cement replacement by metakaolin reaching to 12%. In this study, the increases between mixes are insignificant; however, it should be noted that further exploration is required especially when the spacing between reinforcement bars reaches 41 mm.

Although the 50% glass replacement as a fine aggregate would perform well and provide higher values of L-box ratios than the control mix replacing cement with metakaolin reduces the L-box ratios at a rate of 11.7%. Srikanth and Lalitha [23] confirmed Singh and Siddique [24] studies’ results and reported the highest L-box ratios which were at the mix where the GP replacement reached 40% of the cement regardless of the binder materials used as partial cement replacement from fly ash or alccofine. Thus, though confirmed that using GWP as fine aggregate would enhance the fresh properties of SCC while adding or partially replacing cement with CWP would significantly impact the fresh properties and reduce it even though if GWP was partially replacing the fine aggregate at low or high levels (10 or 15%).

### 6.2. Hardened State Properties

As stated earlier, three strength tests evaluate the hardened properties in this study. The results were obtained and compared by the EGP 203 [29] as follows:

#### 6.2.1. Compressive Strength

**Figure 16:**
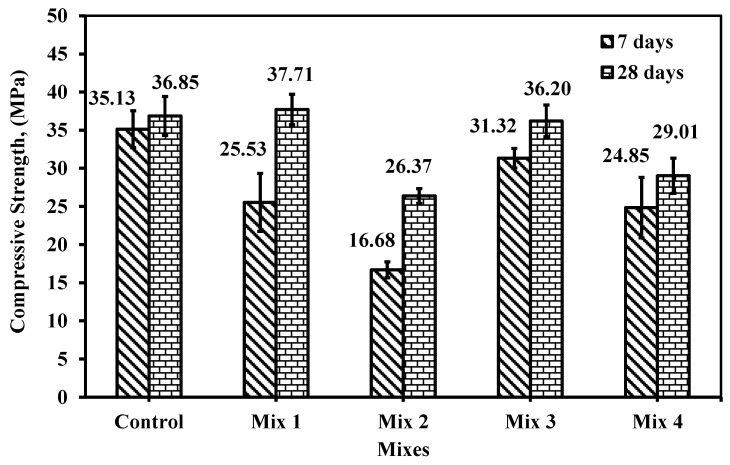
Compressive strength of cube specimens for the five mixes at 7 and 28 days.

Figure 16 shows below the compressive strength of 7 and 28 days of age with different dosages of CWP and RG applied through mixes. The standard deviation indicates how reliable is the data and uniform results among specimens. As shown in Figure 16, the control mix dominates the compressive strength above the other mixes at 7 days of age. This behavior is attributed to the CWP which acts as low heat precursor as it contains a similar composition to metakaolin. Thus, at 28 days, it is expected to see high development in strength which is clear having assumed the CWP was not working only as crystalline filler but also might have reacted while the hydration process between four cement components and water took place. These findings are aligned with Chen et al. [16] who studied the utilization of CWP as partial cement replacement and found that the compressive strength was affected by replacing the cement with CWP. For instance, they reported that the increase in CWP content would reduce the early compressive strength. Further, they confirmed that at a later age of 56 days with CWP ranges between 10 and 20% would be achieving the appropriate compressive strength over its competition the control. Similar findings were deduced by El-Dieb et al. [15] who stated a concrete of grade 25 MPa could be produced by 10 to 20% CWP replacement without any significant influence on strength. For further increase, the early strength would be influenced by 18%. Here in this study, the compressive strength of the cube specimen at 7 days relevant to the control would be reduced by 27.33, 52.32, 10.85, and 29.27% for mix 1, 2, 3, and 4, respectively. At 28 days of age, the percentage of reduction in the same case was lowered showing the gain and developing of strength at a later age. For instance, while mixes 2, 3, and 4 had a reduction of 28.44, 1.8 and 21.28%, respectively, only mix 1 showed enhancement compared to the reference mix by 2.33% although the reduction was about at 7 days which the cement replacement was 20% of cement and 5%only of fine aggregate was replaced by recycled glass although El-Dieb et al. [15] confronted that partial cement replacement till 30% would provide a concrete of grade 25 MPa which the case in this study specifically mix 4.

On the other hand, the development of control, mixes 1, 2, 3, and 4 from 7 to 28 days of age were 95.33, 67.71, 63.52, 86.52 and 85.66%, respectively. Nevertheless, as most of the mixes were partial cement replacement by 20, 15, and 25% CWP, the glass replacement by fine aggregate plays an important role in compressive strength. In mix 1, the compressive strength reached that of control as the cement partial replacement was 20%, which most researchers [12,13,14,15,16,17] agreed to be the optimal value to achieve similar strength of the reference mixtures. In this study, mix 1 also has 5% of glass replacement by fine aggregate which confirms that 5% would not influence the strength. This behavior does not comply with findings that, as the RG was added as a replacement to fine aggregate, the strength would increase. However, increasing the RG as in mix 2 reduced the compressive strength by 52.32 and 28.44% at 7 and 28 days of age. The reduction while replacing 15% RG was very high at 7 days than that of mix 1 by about 35%. Similarly, at 28 days, the reduction reached about 30% as a result of employing 15% RG as a fine replacement. This behavior changed totally in mixes 3 and 4 while RG was kept constant at 10% fine aggregate replacement. The CWP was changed to 15 and 25%.

In the existing literature [12,13,14], the CWP was set to a range of 10 to 20% only that would not influence the compressive strength negatively. Hence, when the CWP is reduced to 1%, then the reduction in strength would be attributed to 10% RG replacement of fine aggregate as in mix 3. Nevertheless, in mix 4, it was found that the reduction increased again although the RG was kept constant at 10%, the CWP was the influence when increased to 25%, which complies with the optimum values amended by previous researchers [14,15,16,17]. Comparing mix 1 and 3 with the control the compressive strength yielded a nearly similar strength with a minor margin that could be negligible. Mix 1 mainly replaced partial cement replacement by 20%, while mix 3 reached 15% with a difference in glass replacement of 5% higher than in mix 1 (5% RG of fine aggregate replacement).

#### 6.2.2. Flexural Strength

**Figure 17:**
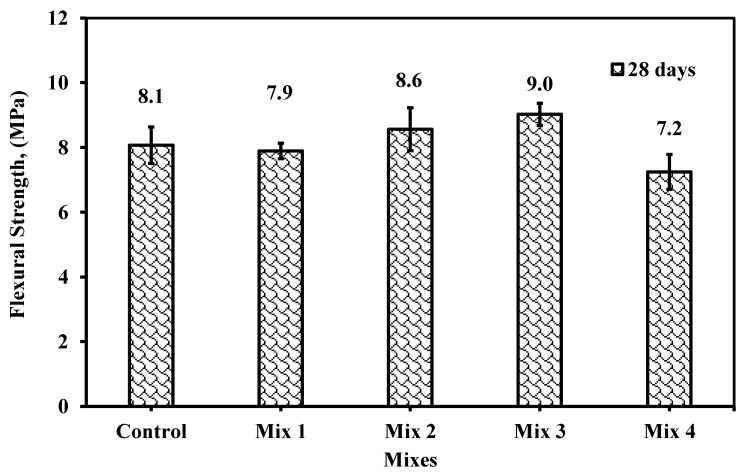
Flexural strength of the prism specimens for the five mixes at 28 days.

Figure 17 provided the results of the flexural strength of prism specimens at 28 days of age. The trend in flexural is similar to that in compressive strength. the flexural performance seems to be higher when fine aggregate was replaced by 15 and 10% of RG. In mix 1, the reduction is very minor to negligible and mainly achieves the same flexural strength while increasing the CWP replacement til 25% and keeping RG by 10%, thus, the reduction by 10.17% would occur. On the contrary, the enhancement of flexural is clear in mix 3 by 11.89%. Similarly, mix 2 has 15% RG and 20% CWP of cement replacement. The enhancement in flexural reached 6.2%, which could be negligible. The reason for the low enhancement relevant to mix 3 is the increase of CWP, which was higher by 5% in mix 2 than in mix 3.

Unfortunately, few researchers have discussed through literature how they would confront this behavior. Singh and Siddique [24] stated that the flexural strength of concrete produced by RG replacement as fine aggregate was reduced, and the only parameter that improved this reduction relevant to the reference is the cement replacement by Mk. The improvement is pronounced in lowering the reduction of the reference mixture, not enhancing the strength of the reference mixture.

Similarly, Ali and Al-Tersawy [37] recorded a reduction in compressive, flexural, and tensile strength along with elastic modulus as the RG content was replaced by the fine aggregate increase. Their explanation was attributed to the SEM investigation in which it was found there is poor contact between the cement matrix and RG. This poor contact was the result of the strength of the weak bond employed in the interfacial transition zone (ITZ) between the cement paste and recycled glass due to the high smoothness of their surfaces. Hence, cracks in ITZ would influence the bond behavior between their surface and cement paste.

#### 6.2.3. Splitting Tensile Strength

**Figure 18:**
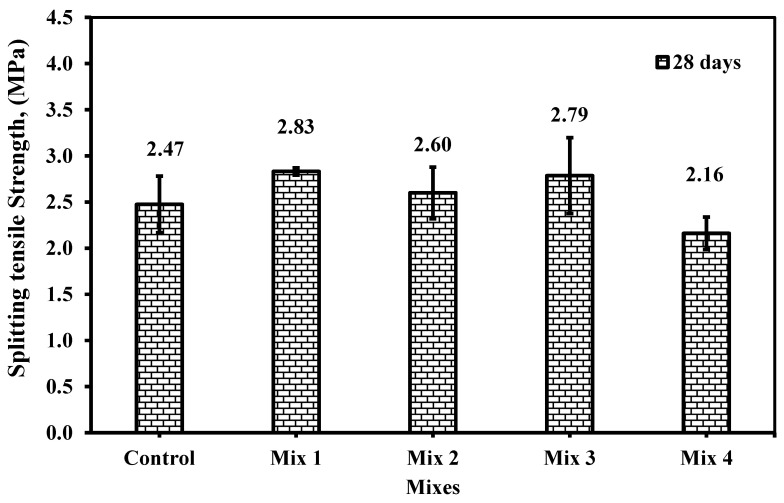
Splitting tensile strength of the cylinder specimens for the five mixes at 28 days.

Splitting tensile strength is a very crucial property, especially when speaking about structures with crack control parameters. Figure 18 provides the splitting tensile strength results of the mixes. The tensile strength showed enhancement in all mixes except mix 4, as shown in Figure 18, to flexural strength relevant to control which complies with the results of flexural strength discussed above. However, the behavior was slightly different, especially since mixes 1, 2, and 3 showed the same trend in flexural with an increase relevant to the control mix by 5.26% and 12.95%, which compiles with the value of enhancement by 6.2 and 11.8% for flexural strength. The same reduction was deduced by mix 4 which was 12.56% for splitting tensile strength and 10.17% for flexural strength. The enhancement of mix 1 is still not understandable and does not comply with any of the references, denoted by how, in this study, the only illustration of this behavior is the micro-filler of CWP and the pozzolanic reactivity which increases or enhances the tensile strength and the bond strength between cement paste and the 5% RG replacement. In other words, the CWP enhanced the ITZ and thus utilized the fiber in the glass with the RG which increased the splitting tensile capacity. A similar explanation to flexural strength is anticipated for mixes 2, 3, and 4.

### 6.3. Relationships Between the Mechanical Strength (Compressive, Flexural, and Tensile)

There is no clear vision for the relationships between strengths for concrete produced via recycled construction wastes yet. Based on the results, which are limited to the five mixes, the relationship between the compressive, flexural, and splitting tensile strengths can be deduced, while utilization of other recycled wastes requires addressing and exploring the existing codes and guidelines that predict flexural and tensile strength. Thus, the empirical percentile estimation was discussed here as a start. The flexural strength could be estimated by around 24.56% on average of compressive strength according to the aforementioned results, while 7.73% on average of compressive strength is needed to determine the splitting tensile strength. These percentiles are above the normal concrete, which is noted for flexural strength by about 15 to 23% of compressive strength, and tensile strength estimated to be 10 to 15%, which here in this study has proven to be less than 10%. Finally, the relation between the flexural and splitting tensile strength does not exceed 23 to 25%; however, here in this study, it was noted as 31.48%.

## 7. Conclusions

This study investigated the possibility of maintaining the same properties of self-compacted concrete while using several replacements composed of recycled wastes, such as CWP and RG, as partial cement and fine aggregate replacement, reducing the environmental impact of the cement and the construction industrial wastes while avoiding deployment of strategic natural resources. Many researchers have suggested the utilization of CWP as a cement replacement and others have suggested the utilization of RG as a partial fine aggregate replacement within traditional normal and self-compacted concrete. Five mixes were suggested utilizing both CWP and RG as cement and fine aggregate replacement at suggested ranges between 10 and 30% for CWP and 5 to 15%. Rheological and mechanical properties were evaluated and the following conclusions were drawn:

(1) A possible effective solution exists for using CWP and RG in SCC manufacturing, reducing the environmental impact of waste deposition, cement, and concrete manufacturing.
(2) The results revealed good rheological properties that comply with code and guidelines, especially ECP 203.
(3) Partial replacement cement with 15 to 20% CWP and fine aggregate by 5 to 10% RG would enhance the flowability due to their hydrophobic properties.
(4) The passing ability of SCC while utilizing the CWP and RG provides privilege in congested reinforcement members.
(5) Generally, the compressive strength is reduced slightly, which is not critical; however, the flexural strength is enhanced, which is a good achievement.
(6) Compressive, flexural, and tensile strengths were reduced as CWP was replaced by more than 20% cement and over 10% RG as a fine aggregate replacement.
(7) The optimum and best performance was achieved at 15% CWP and 10% RG replacement of cement and fine aggregate, especially in flexural and tensile strengths corresponding to the control mix of SCC.

Further recommendations are required to examine the segregation properties and durability of the concrete produced. The investigation should include also the imaging processing and movement between particles while passing through the reinforcement spacing adopted for the project in which SCC with recycled wastes would be utilized, in addition to non-destructive testing for collating the destructive data for Schmidt hammer and Ultrasonic pulse velocity applications.

## Figures and Tables

**Table 1:** Shows the mix design and the portion of SCC ingredients.

Mix Code	Designation	Water (kg/m^3^)	Cement (kg/m^3^)	CWP (kg/m^3^)	CA (kg/m^3^)	FA (kg/m^3^)	RG (kg/m^3^)
Control	100% C + 100% FA	231	550	-	612	909	0
Mix 1	20% CWP + 5% RG	231	440	110	612	863.55	45.45
Mix 2	20% CWP + 15% RG	231	440	110	612	772.65	136.35
Mix 3	15% CWP + 10% RG	231	467.5	82.5	612	818.1	90.9
Mix 4	25% CWP + 10% RG	231	412.5	137.5	306	818.1	90.9

CWP: Ceramic Waste Powder; CA: Coarse Aggregate; FA: Fine Aggregate; RG: Recycled Glass.

**Table 2:** Shows the chemical composition [27].

Materials	Cement	Ceramic Powder (CWP)	Glass (RG)
SiO_2_	25.3	63.9	81.98
Al_2_O_3_	6.64	18.29	0.86
Fe_2_O_3_	6.68	4.32	0.23
CaO	58.44	4.46	10.67
MgO	2.29	0.72	5.63
P_2_O_5_	0	0.16	0.12
K_2_O	0.25	2.18	0.23
Na_2_O	0.66	0.75	-
SO_3_	2.04	0.1	0.19
Cl	0.06	0.005	-
TiO_2_	-	0.61	-
SrO_2_	-	0.02	-
Mn_2_O_3_	-	0.05	-
LOI	4	1.61	-

**Table 3:** Upper and lower limits of the rheological tests on SCC as per ECP 203 [29].

The Rheological Test	Units	Limits	
		Min.	Max.
Slump Flow (diameter)	mm	600	800
Time for reaching slump flow with a diameter of 500 mm (T_50 cm_)	sec	2	5
J-ring slump flow (diameter)	mm	0	20
V-funnel after immediate mixing (t_o_)	sec	6	12
V-funnel after 5 min from mixing (t_5 min._)	sec	t_o_	t_o_ + 3
L-box (H_2_/H_1_)	ratio	0.80	1.0

## Data Availability

The original contributions presented in the study are included in the article, further inquiries can be directed to the corresponding author.
